# Intrinsic exercise capacity is associated with skeletal muscle clock gene and IGF-1 signaling in aged low- and high-running capacity rats

**DOI:** 10.3389/fphys.2026.1818866

**Published:** 2026-06-09

**Authors:** Hyeon-Ki Kim, Takuji Kawamura, Zoltan Bori, Lauren Gerard Koch, Steven Loyal Britton, Zsolt Radak

**Affiliations:** 1 Research Center for Molecular Exercise Science, Hungarian University of Sport Science, Budapest, Hungary; 2 Medicine and Science in Sports and Exercise, Graduate School of Medicine, Tohoku University, Sendai, Japan; 3 Center for Physical Activity Research, National Institutes of Biomedical Innovation, Health and Nutrition, Osaka, Japan; 4 Department of Public Health and Health Policy, Graduate School of Biomedical and Health Sciences, Hiroshima University, Hiroshima, Japan; 5 Smart-Aging Research Center, Tohoku University, Sendai, Japan; 6 Department of Physiology and Pharmacology, The University of Toledo College of Medicine and Life Sciences, Toledo, OH, United States; 7 Department of Anesthesiology, University of Michigan, Ann Arbor, MI, United States; 8 Institute of Sport Sciences and Physical Education, Faculty of Sciences, University of Pécs, Pécs, Hungary; 9 Department of Bioengineering, Sapientia Hungarian University of Transylvania, Miercurea Ciuc, Romania

**Keywords:** aging, circadian clock genes, IGF-1 signaling, intrinsic exercise capacity, low- and high-capacity runner rats, skeletal muscle

## Abstract

**Background:**

Intrinsic exercise capacity is a strong predictor of health and longevity and is independently associated with aging- and disease-related outcomes. Although previous studies using low- and high-capacity runner (LCR and HCR) rats have demonstrated organ-specific patterns of epigenetic aging, the molecular mechanisms linking intrinsic aerobic capacity to skeletal muscle signaling remain incompletely understood.

**Objective:**

This study investigated whether intrinsic exercise capacity is associated with alterations in skeletal muscle clock gene expression and insulin-like growth factor-1 (IGF-1)–related signaling pathways.

**Methods:**

Female LCR and HCR rats (23–24 months old, 44th generation) underwent maximal oxygen uptake (VO_2_max) testing, followed by molecular analyses of plantaris and soleus muscles using quantitative PCR and Western blotting.

**Results:**

VO_2_max was significantly higher in HCR rats. In skeletal muscle, LCR rats exhibited higher mRNA expression levels of Cry1, Bmal1, Igf1, and Igf1Ec measured at ZT2–ZT3, whereas expression of Cry2, Pdk4, and MuRF-1 did not differ between phenotypes. Despite increased *Igf1* expression in LCR rats, the phosphorylated-to-total JAK2 ratio was reduced, while STAT5 phosphorylation was unchanged. Correlation analyses demonstrated significant negative associations between VO_2_max and *Igf1*, *Igf1Ec*, *Cry1*, and *Bmal1* expression.

**Conclusion:**

These findings indicate that low intrinsic exercise capacity is associated with coordinated alterations in skeletal muscle clock gene expression and IGF-1– related signaling, suggesting altered IGF-1–related signaling balance in aging skeletal muscle. These results provide mechanistic insight into how intrinsic aerobic capacity may influence muscle biology and health trajectories during aging.

## 1 Introduction

Intrinsic exercise capacity is strongly associated with health and longevity and has been reported to predict mortality risk independently of traditional cardiometabolic factors (Blair et al., 1989; Myers et al., 2002; Wisløff et al., 2005). In both human and animal studies, low aerobic capacity is strongly associated with increased susceptibility to metabolic disorders, cardiovascular disease, and reduced lifespan (Koch and Britton, 2001; Myers et al., 2002). To investigate the biological mechanisms underlying these associations, selectively bred low- and high-capacity runner (LCR and HCR) rats have been established as a robust experimental model of intrinsic aerobic fitness (Koch and Britton, 2001).

Using this model, recent work has reported associations between intrinsic exercise capacity and systemic aging phenotypes, including organ-specific patterns of epigenetic aging (Kawamura et al., 2025). These findings suggest that aerobic capacity may be linked to broader regulatory processes involved in aging biology. However, while epigenetic profiling provides insight into global regulatory alterations, it does not fully explain how intrinsic exercise capacity is translated into functional molecular phenotypes within metabolically active tissues.

Skeletal muscle plays an important role in exercise performance and whole-body metabolic regulation (Bengt and Philip, 1983). Alterations in skeletal muscle metabolic programming have been implicated in insulin resistance, sarcopenia, and age-related functional decline (Bengt and Philip, 1983; Glass, 2005). However, the molecular mechanisms through which intrinsic aerobic capacity may be reflected in skeletal muscle remain incompletely understood.

Accumulating evidence indicates that circadian clock genes contribute to skeletal muscle metabolic regulation and exercise adaptation (Martin et al., 2023). Core clock components, including cryptochromes (*Cry1* and *Cry2*) and brain and muscle ARNT-like protein 1 (*Bmal1*), regulate mitochondrial function, substrate utilization, and metabolic flexibility (Jordan et al., 2017; Martin et al., 2023). Notably, cryptochromes have been reported to repress peroxisome proliferator–activated receptor delta (PPARδ) signaling, thereby influencing endurance capacity and oxidative metabolism (Jordan et al., 2017). However, whether skeletal muscle clock gene expression differs according to intrinsic aerobic capacity in aged LCR/HCR rats remains unclear.

Insulin-like growth factor-1 (IGF-1) signaling axis is a major regulator of skeletal muscle growth, metabolism, and aging-related processes (Glass, 2005; Junnila et al., 2013). Appropriate regulation of IGF-1 signaling is essential for maintaining physiological homeostasis, as both insufficient and excessive signaling may have adverse consequences (Junnila et al., 2013). Altered regulation of IGF-1–related pathways has been implicated in age-related muscle dysfunction and chronic metabolic diseases (Glass, 2005; Junnila et al., 2013). Moreover, accumulating evidence suggests interactions between circadian clock components and growth-related signaling pathways, indicating that these systems may converge to shape muscle function and aging trajectories (Jordan et al., 2017).

Therefore, the present study aimed to examine whether intrinsic exercise capacity is associated with differences in skeletal muscle clock gene expression and IGF-1–related signaling pathways in aged female LCR and HCR rats.

## 2 Methods

### 2.1 Animals

Female low- and high-capacity runner rats (LCR and HCR) from the 44th generation were used in the present study. All animals were 23–24 months of age at the time of experimentation. A total of 19 rats were included (HCR, n = 10; LCR, n = 9). Rats were housed under controlled environmental conditions (12:12-h light–dark cycle, temperature 22 ± 2 °C, relative humidity approximately 55 ± 10%) with ad libitum access to standard laboratory chow and water. All experimental procedures were conducted in accordance with the European Directive 2010/63/EU for animal experiments and were approved by the National Animal Research Ethical Committee of Hungary (approval no. PE/EA/62-2/2021).

### 2.2 Maximal oxygen uptake measurement

Maximal oxygen uptake (VO_2_max) was assessed using a motor-driven treadmill system in a closed metabolic chamber customized for rats (Columbus Instruments, USA), following the same methodology as in previous studies of the LCR/HCR model. Briefly, all rats were acclimated to treadmill running prior to VO_2_max measurements. Animals rested on the running belt for 5 min, after which running speed was gently increased. Acclimatization runs were performed for 5–10 min at speeds ranging from 5 to 25 m/min on two consecutive days. For VO_2_max testing, rats began running at 5 m/min following a 5-min rest period, and treadmill speed was increased by 5 m/min every 2 min until exhaustion. VO_2_max was determined for each animal when at least one of the following criteria was met:

- no further increase in oxygen uptake despite increasing running speed;
- inability to maintain posture on the treadmill;
- respiratory quotient (RQ = VCO_2_/VO_2_) exceeding 1.0.

VO_2_max values were recorded when any of the predefined exhaustion criteria were reached. All VO_2_max measurements were conducted during the light phase under controlled environmental conditions (22 ± 2 °C, relative humidity approximately 55 ± 10%). Measurements were performed between approximately ZT1 and ZT7 for both HCR and LCR rats. Although testing was conducted across a relatively broad time window, measurement timing and experimental conditions were coordinated between groups to minimize potential circadian-related variability.

### 2.3 Tissue collection and circadian timing

Skeletal muscle tissues (plantaris and soleus) were collected at least 48 hours after completion of the VO_2_max measurements under deep anesthesia, rapidly excised, cleared of visible connective tissue, snap-frozen in liquid nitrogen, and stored at −80 °C until analysis. All dissections were performed during the light phase, with Zeitgeber time defined relative to lights on (ZT0), corresponding approximately to ZT2–ZT3; sampling times showed substantial overlap between HCR and LCR rats and were restricted to a narrow time window to limit potential circadian influences on clock gene expression.

### 2.4 RNA extraction and quantitative real-time PCR

Total RNA was extracted from plantaris and soleus muscles using the RNeasy Mini Kit (Qiagen, Hilden, Germany) according to the manufacturer’s instructions. Reverse transcription was performed using the Maxima First Strand cDNA Synthesis Kit (Thermo Fisher Scientific, Waltham, MA, USA), using standardized amounts of total RNA (0.5–1 μg) for each sample to minimize variability in cDNA synthesis efficiency. Quantitative real-time PCR (qRT-PCR) was performed using the LightCycler 480 SYBR Green I Master mix (Roche, Basel, Switzerland). Each reaction was carried out in a total volume of 20 μL containing 10 μL of 2× SYBR Green master mix, 1 μL of cDNA template, gene-specific primers (final concentration 0.3 μM each), and nuclease-free water. Amplification was performed using the QuantStudio 3/5 Real-Time PCR System (Thermo Fisher Scientific). All samples were analyzed in technical triplicates. Melting curve analysis was performed to confirm amplification specificity. Mean Ct values were used for subsequent analysis. Relative gene expression levels were calculated using the 2^−ΔΔCt method, with ribosomal protein L13 (RPL13) serving as the housekeeping gene. Expression levels were normalized to RPL13 and expressed relative to the HCR group.

Primer sequences
RPL13
Forward: CTTGAGGCTAAGGAAACAGG   
Reverse: ACAGTCTTTATTGGGTTCACAC
*MuRF-1*
Forward: CTCCTTGTGCAAGGTGTTCG   
Reverse: TGCTCAGTTCAGTCTTCTGTCC
*Atrogin-1*
Forward: CAGCCTGAACTACGATGTTGC   
Reverse: CATGGCGCTCCTTAGTACTCC
*Bmal1*
Forward: CAATGCGATGTCCCGGAAGTTAGA   
Reverse: TCCCTCGGTCACATCCCTGAGAAT
*Cry1*
Forward: GTGGTGGCGGAAACTGCTCTC   
Reverse: ACTCTGTGCGTCCTCTTCCTGA
*Cry2*
Forward: GTGTGAATGCAGGCAGCTG   
Reverse: ACAGGGCAGTAGCAGTGGAA
*Pdk4*
Forward: TTCACACCTTCACCACATGC   
Reverse: AAAGGGCGGTTTTCTTGATG
*Ucp3*
Forward: GTGACCTATGACATCATCAAGGA   
Reverse: GCTCCAAAGGCAGAGACAAAG
*IGF-1*
Forward: CTTTACCAGCTCGGCCACA   
Reverse: TTGGTCCACACACGAACTGAAG
*IGF-1Ec*
Forward: TCCGCTGCAAGCCTACAAAGTC  
Reverse: CTTTCCTTCTCCTTTGCAGCTTC

### 2.5 Protein extraction and western blotting

Protein extraction and immunoblotting were performed as described previously (Zhou et al., 2025). Briefly, skeletal muscle samples were homogenized in ice-cold lysis buffer supplemented with protease and phosphatase inhibitors. Protein concentrations were determined using the BCA assay. Equal amounts of protein were separated by SDS–PAGE and transferred onto PVDF membranes. Membranes were incubated with the following primary antibodies: total STAT5A/B Proteinteich, Cat. No. 12071-1-AP]), phospho-STAT5A/B (Tyr694/699; AbboMax : 500-9334]), total JAK2 (Cell Signaling Technology: (D2E12) #3230), phospho-JAK2 (Tyr1007/Tyr1008): bs-2485R]), and PPARδ ([Proteintech: Cat No. 60193-1-Ig]).

**Figure 1:**
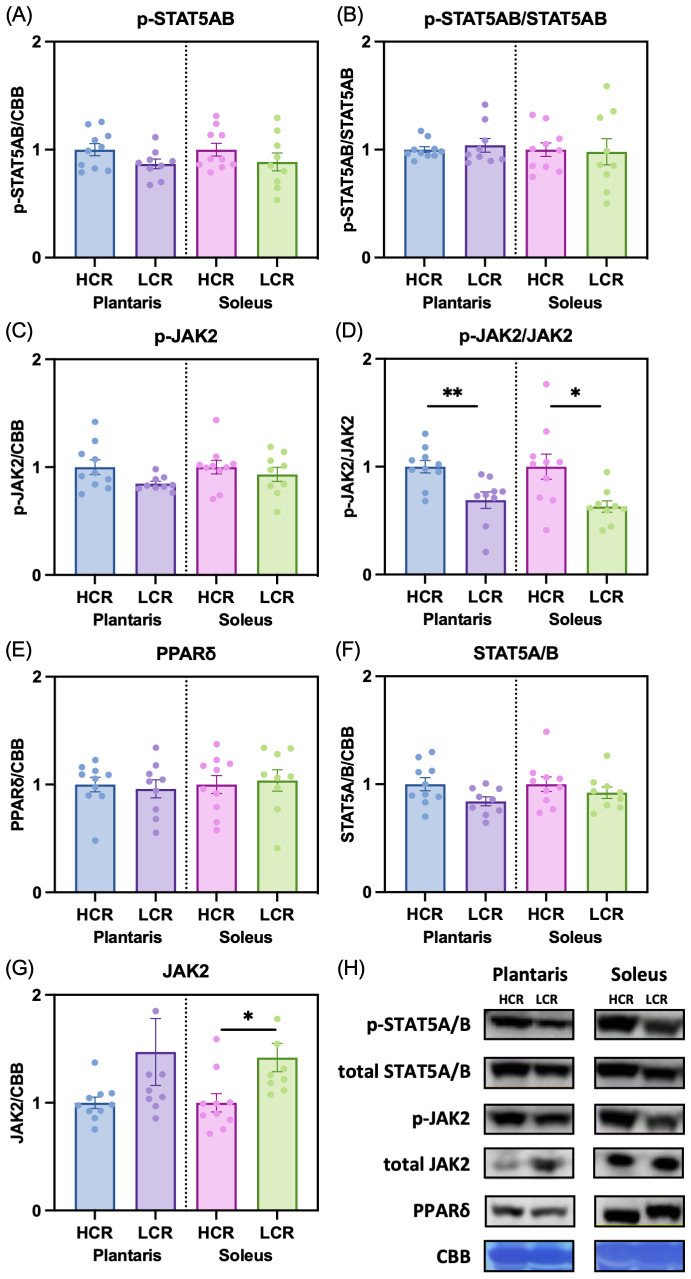
Skeletal muscle IGF-1–related signaling protein expression in aged HCR and LCR rats. Representative immunoblots and quantitative analyses of protein expression in plantaris and soleus muscles of HCR and LCR rats for **(A)** phosphorylated STAT5A/B (p-STAT5A/B), **(B)** p-STAT5A/B normalized to total STAT5A/B, **(C)** phosphorylated JAK2 (p-JAK2), **(D)** p-JAK2 normalized to total JAK2, **(E)** total PPARδ, **(F)** total STAT5A/B, and **(G)** total JAK2 protein expression. Phosphorylated protein levels were normalized to their corresponding total protein levels. Coomassie Brilliant Blue (CBB) staining was used to verify total protein loading and transfer efficiency. Data are presented as individual values with mean ± SD. Statistical comparisons between groups were performed using unpaired two-tailed Student’s t-tests. *p < 0.05, **p < 0.01 versus HCR.

For phosphorylated proteins, membranes were first probed with antibodies against the phosphorylated form, then stripped and reprobed for the corresponding total protein. Band intensities were quantified by densitometry using ImageJ. Total protein loading was assessed by Coomassie Brilliant Blue (CBB) staining of the PVDF membrane. Phosphorylated STAT5A/B and phosphorylated JAK2 signals were normalized to their corresponding total protein levels (STAT5A/B and JAK2, respectively) for calculation of phosphorylation ratios. Total protein loading and transfer efficiency were additionally verified by Coomassie Brilliant Blue (CBB) staining of PVDF membranes. Representative immunoblots and quantitative analyses are shown in **Figure 1**.

### 2.6 Statistical analysis

All data are presented as mean ± standard deviation (SD), with individual data points shown where appropriate. Normality of data distribution was assessed using the Shapiro–Wilk test. As all variables met the assumption of normality, comparisons between high-capacity runner (HCR) and low-capacity runner (LCR) rats were performed using unpaired two-tailed Student’s *t*-tests. Associations between intrinsic aerobic capacity (VO_2_max) and skeletal muscle molecular markers (*Igf1*, *Igf1Ec*, *Cry1*, and *Bmal1*) were evaluated separately for plantaris and soleus muscles using Pearson correlation coefficients. Correlation results are presented as heat maps depicting correlation coefficients and corresponding significance levels. All statistical analyses were conducted using IBM SPSS Statistics for Mac (version 28.0; IBM Corp., Armonk, NY, USA). A two-tailed *P* value < 0.05 was considered statistically significant.

## 3 Results

### 3.1 Phenotypic characteristics of HCR and LCR rats

**Figure 2:**
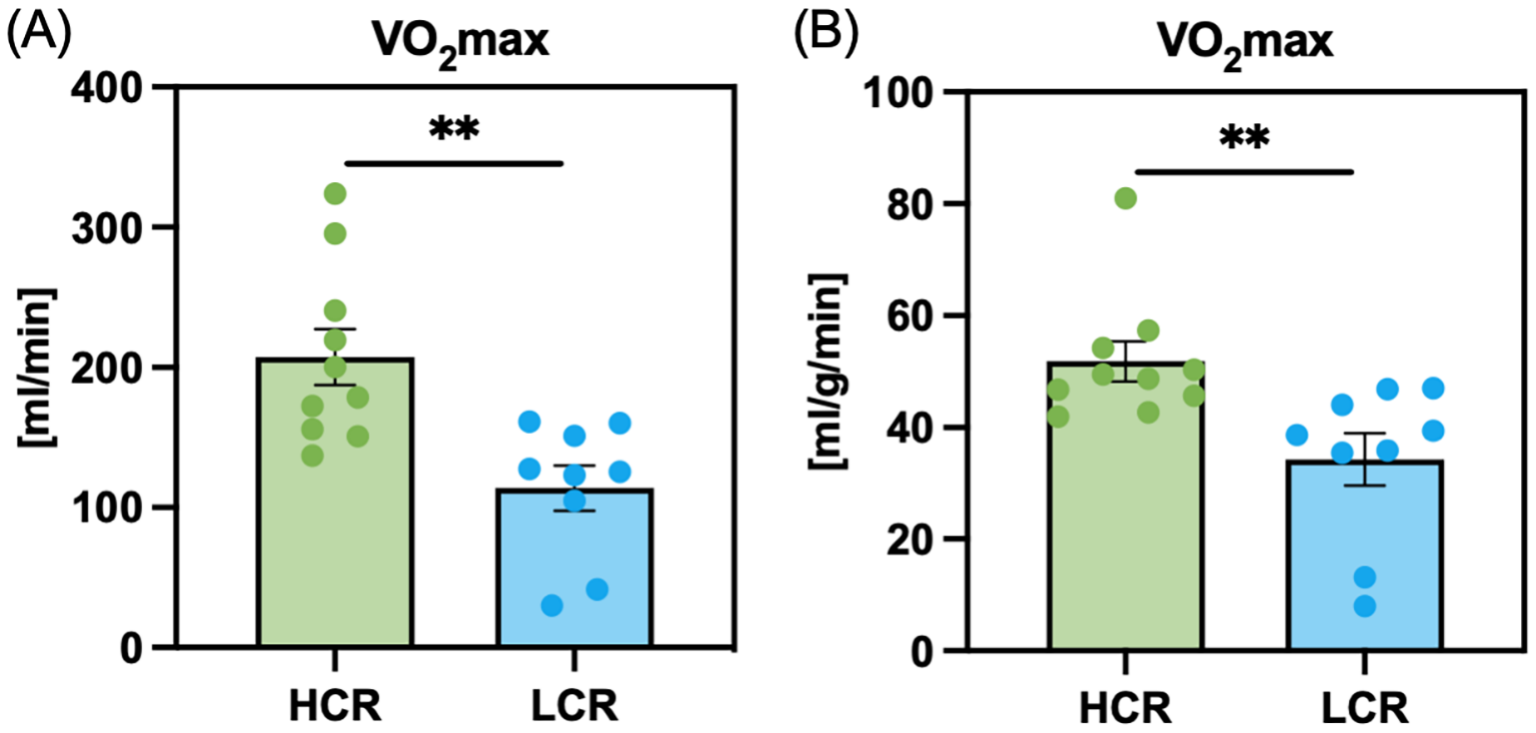
Intrinsic aerobic exercise capacity in high- and low-running capacity rats. **(A)** Absolute maximal oxygen consumption (VO_2_max; mL/min) and **(B)** body mass–normalized VO_2_max (mL/g/min) in high-capacity runner (HCR) and low-capacity runner (LCR) rats. Data are presented as individual values with mean ± SD. Statistical comparisons between groups were performed using unpaired two-tailed Student’s t-tests. **p < 0.01 versus HCR.

Body weight was significantly higher in LCR rats than in HCR rats (p = 0.041; **Supplementary Figure 1**). Intrinsic aerobic exercise capacity, assessed by maximal oxygen consumption (VO_2_max), was significantly higher in HCR rats than in LCR rats (**Figures 2A, B**). This difference was evident when VO_2_max was expressed both as an absolute value (ml/min, p = 0.002; **Figure 2A**) and when normalized to body mass (ml/g/min, p = 0.008; **Figure 2B**), confirming a robust separation of intrinsic aerobic capacity between the two phenotypes.

### 3.2 Skeletal muscle gene expression profiles differ between HCR and LCR rats

**Figure 3:**
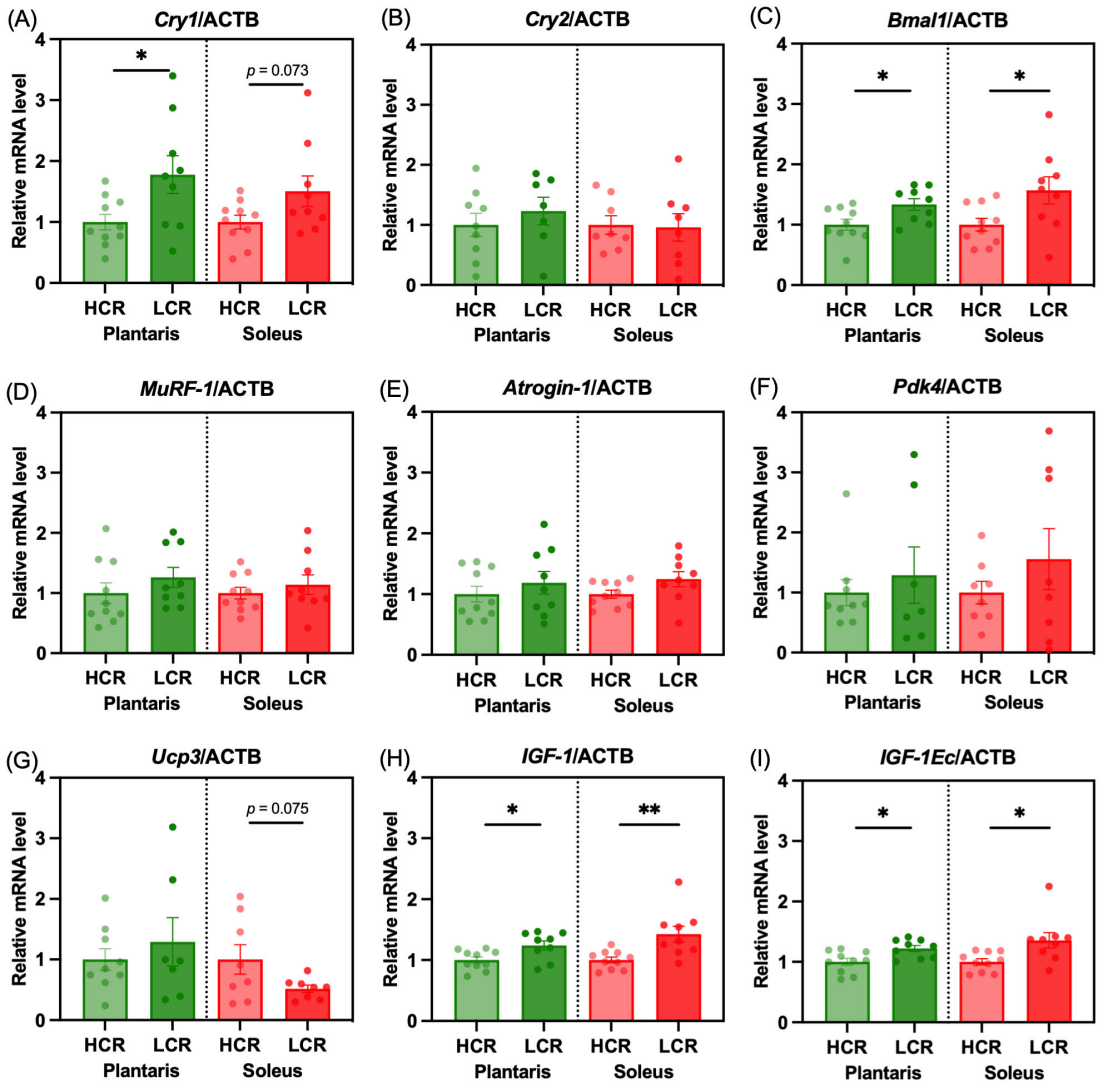
Skeletal muscle gene expression profiles in HCR and LCR rats. Relative mRNA expression levels of clock genes [**(A)** *Cry1*, **(B)** *Cry2*, **(C)** *Bmal1*], the muscle atrophy–related gene [**(D)** *MuRF-1*, **(E)** *Atrogin-1*], metabolic regulators [**(F)** *Pdk4*, **(G)** *Ucp3*], and IGF-1–related genes [**(H)** *Igf1*, **(I)** *Igf1Ec*] in the plantaris and soleus muscles of HCR and LCR rats. Gene expression levels were normalized to RPL13 and expressed relative to the HCR group. Data are shown as individual values with mean ± SD. Statistical comparisons between groups were performed using unpaired two-tailed Student’s t-tests. *p < 0.05, **p < 0.01 versus HCR.

Quantitative PCR analyses revealed distinct skeletal muscle gene expression patterns between HCR and LCR rats in both the plantaris and soleus muscles (**Figure 3**). Among clock genes, *Cry1* mRNA expression was significantly higher in LCR rats than in HCR rats in the plantaris muscle (p = 0.027; **Figure 3A**), with a similar tendency observed in the soleus muscle (p = 0.073; **Figure 3A**). *Bmal1* expression was significantly elevated in LCR rats in both the plantaris (p = 0.021) and soleus muscles (p = 0.030; **Figure 3C**), whereas *Cry2* expression did not differ significantly between phenotypes in either muscle (**Figure 3B**). Expression of the muscle atrophy–related genes *MuRF-1* and *Atrogin-1* did not differ significantly between phenotypes (**Figures 3D, E**). Genes related to metabolic regulation showed muscle-specific responses. *Pdk4* expression did not differ significantly between HCR and LCR rats (**Figure 3F**), while *Ucp3* expression exhibited a trend toward lower expression in the soleus muscle of LCR rats (p = 0.075; **Figure 3G**). In contrast, components of the IGF-1 pathway were consistently altered. *Igf1* mRNA expression was significantly higher in LCR rats in both the plantaris (p = 0.017) and soleus muscles (p = 0.005; **Figure 3H**), with a more pronounced difference in the soleus muscle. Similarly, *Igf1Ec* expression was significantly higher in LCR rats than in HCR rats in both muscles (plantaris: p = 0.011; soleus: p = 0.016; **Figure 3I**).

### 3.3 Altered IGF-1–related signaling at the protein level in skeletal muscle

Western blot analyses demonstrated phenotype-dependent differences in IGF-1–related signaling pathways in skeletal muscle (**Figure 1**). Phosphorylated STAT5A/B levels, total STAT5A/B protein abundance, and the ratio of phosphorylated to total STAT5A/B did not differ significantly between HCR and LCR rats in either muscle (**Figures 1A, B, F**). In contrast, total JAK2 protein abundance was higher in the soleus muscle of LCR rats than in HCR rats (**Figure 1G**, p = 0.014). Nevertheless, the ratio of phosphorylated JAK2 to total JAK2 was significantly lower in LCR rats compared with HCR rats in both plantaris (p = 004; **Figure 1D**) and soleus muscles (p = 0.014; **Figure 1D**), indicating altered JAK2-related signaling balance. Total PPARδ protein expression did not differ significantly between phenotypes in either muscle (**Figure 1E**).

### 3.4 Associations between VO_2_max and skeletal muscle molecular markers

**Figure 4:**
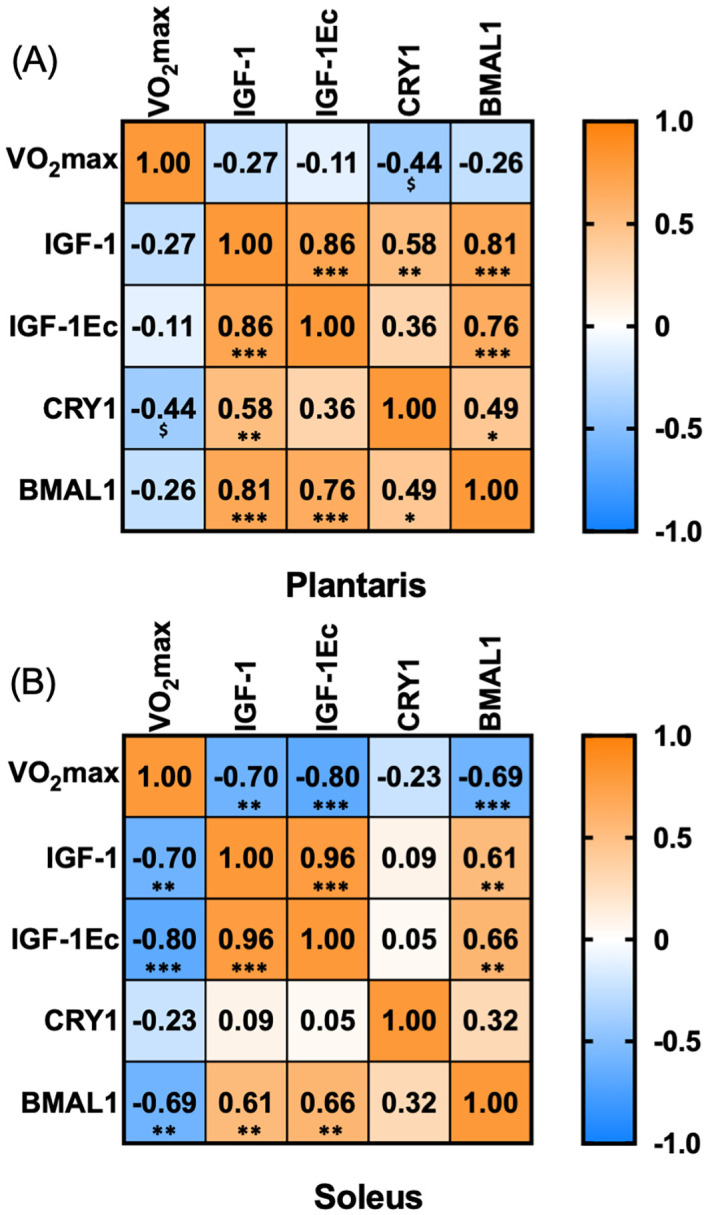
Associations between intrinsic aerobic capacity and skeletal muscle molecular markers.Heat maps showing Pearson correlation coefficients between VO_2_max and skeletal muscle gene expression levels in plantaris and soleus muscles of HCR and LCR rats. Correlations are shown for **(A)** plantaris muscle and **(B)** soleus muscle. Variables include Igf1, Igf1Ec, Cry1, and Bmal1 expression levels. Color scale represents the strength and direction of correlations (−1 to +1). Statistical significance is indicated within each panel.

Correlation analyses revealed significant associations between intrinsic aerobic capacity and skeletal muscle molecular markers (**Figure 4**). In the plantaris muscle, VO_2_max was negatively correlated with *Igf1*, *Igf1Ec*, *Cry1*, and *Bmal1* expression, whereas *Igf1* and *Igf1Ec* were strongly positively correlated with *Bmal1* expression. *Cry1* expression also showed moderate positive correlations with IGF-1–related markers (**Figure 4A**). In the soleus muscle, VO_2_max exhibited stronger negative correlations with *Igf1* and *Igf1Ec* expression, while positive correlations were observed between *Igf1*/*Igf1Ec* and *Bmal1* expression. Associations between *Cry1* and IGF-1–related markers were weaker in the soleus compared with the plantaris muscle (**Figure 4B**).

## 4 Discussion

The present study shows that intrinsic exercise capacity is associated with distinct skeletal muscle molecular profiles in aged female LCR and HCR rats. In particular, low intrinsic aerobic capacity was characterized by elevated expression of clock genes (*Cry1* and *Bmal1*) and IGF-1–related transcripts, accompanied by reduced JAK2 phosphorylation. These findings suggest differences in growth-related and circadian-associated signaling pathways between phenotypes.

Previous investigations using the LCR/HCR model have reported associations between intrinsic exercise capacity and cardiometabolic risk, lifespan, skeletal muscle metabolic function, and organ-specific patterns of aging (Koch and Britton, 2001; Wisløff et al., 2005; Kawamura et al., 2025). Previous studies have demonstrated that LCR rats exhibit impaired skeletal muscle metabolism, reduced mitochondrial respiratory capacity, altered energy metabolism–related metabolite profiles, and increased disease susceptibility compared with HCR rats (Kivelä et al., 2010; Lessard et al., 2011; Stephenson et al., 2012). The greater body weight observed in LCR rats in the present study is consistent with previous reports using the HCR/LCR model and is considered part of the co-segregating phenotype associated with low intrinsic aerobic capacity, metabolic dysfunction, and increased disease susceptibility (Koch and Britton, 2001; Wisløff et al., 2005). Although VO_2_max was evaluated using both absolute and body mass–normalized values, differences in body mass may still influence metabolic and physiological outcomes and should therefore be considered when interpreting the present findings. In addition, aging and exercise training have been shown to differentially influence skeletal muscle metabolic regulation between LCR and HCR phenotypes (Shi et al., 2018; Zhuang et al., 2021). The present findings extend these observations by identifying associations between intrinsic aerobic capacity and skeletal muscle clock gene transcript expression as well as IGF-1–related signaling balance in aged animals.

A central finding of this study was the differential expression of selected skeletal muscle clock gene transcripts between low- and high-capacity phenotypes. Core clock components, including *Cry1/2* and *Bmal1*, have been implicated in skeletal muscle metabolism and exercise-related physiological processes, suggesting that altered expression of selected clock gene transcripts may be associated with differences in skeletal muscle metabolic regulation. These findings suggest that altered expression of selected clock gene transcripts may be associated with differences in skeletal muscle metabolic regulation in aged LCR and HCR rats.

One notable observation of the present study was the differential expression of selected skeletal muscle clock gene transcripts between HCR and LCR rats. In particular, *Cry1* and *Bmal1* expression levels measured at ZT2–ZT3 were higher in LCR rats than in HCR rats. Circadian regulators have been implicated in skeletal muscle metabolism and exercise-related physiological processes (Ahmad et al., 2020). Therefore, the present findings suggest that altered expression of skeletal muscle clock genes may be associated with differences in metabolic regulation between low- and high-capacity phenotypes.

Furthermore, the observed differences in IGF-1–related signaling pathways suggest that skeletal muscle may contribute to the integration of intrinsic exercise capacity with processes associated with aging and disease susceptibility. Tight regulation of IGF-1 signaling is essential for maintaining physiological homeostasis, and alterations in the balance of this axis have been linked to metabolic disorders and age-related functional decline (Junnila et al., 2013; Chaudhari et al., 2017). In the present study, increased *Igf1* and *Igf1Ec* transcript expression in LCR rats was accompanied by reduced JAK2 phosphorylation and unchanged STAT5 activation, suggesting a potential imbalance in IGF-1–related signaling. One possible interpretation is that elevated *Igf1* expression represents a compensatory response to impaired downstream signaling responsiveness in aged skeletal muscle. Alternatively, reduced JAK2 phosphorylation despite increased *Igf1* transcript abundance may reflect altered receptor–JAK2 coupling efficiency or inhibitory regulatory mechanisms associated with aging and low intrinsic exercise capacity. Because the present study assessed signaling markers at a single time point without direct functional manipulation, these interpretations remain speculative and warrant further mechanistic investigation. These findings may reflect complex age-related alterations in anabolic signaling regulation associated with low intrinsic exercise capacity.

### 4.1 Clock gene signaling and intrinsic exercise capacity

One important finding of the present study is that skeletal muscle clock gene signaling was associated with intrinsic exercise capacity, independent of exercise training. Circadian clock genes are known for regulating circadian rhythms, but they have also been shown to play a fundamental role in determining baseline metabolic and functional capacity (Dyar et al., 2014). In skeletal muscle, clock components such as *Cry1/2* and *Bmal1* influence mitochondrial function, substrate utilization, and oxidative metabolism, processes that are closely linked to endurance performance (Andrews et al., 2010). In the present study, we observed differences in clock gene-related signaling between low- and high-running capacity phenotypes, indicating that intrinsic aerobic capacity is linked to the circadian clock-regulating network in skeletal muscle. Given that cryptochromes have been reported to repress PPARδ-related metabolic activity and downstream signaling pathways (Jordan et al., 2017), altered *Cry1/2* expression may be associated with modified metabolic regulation rather than altered total PPARδ protein abundance. This interpretation is consistent with prior studies demonstrating that suppression of PPARδ signaling can limit oxidative capacity and endurance performance (Wang et al., 2004; Narkar et al., 2008). These findings broaden our understanding of clock gene function, suggesting that circadian rhythmic control mechanisms contribute to a molecular framework related to intrinsic exercise capacity and metabolic control. However, the present data are correlative, and more research is needed to understand how circadian rhythmic signaling interacts with the genetic background to influence aerobic capacity and metabolic health.

### 4.2 IGF-1 signaling as a link between exercise capacity and aging

This study demonstrated an association between intrinsic exercise capacity and skeletal muscle IGF-1-related signaling. The IGF-1 system plays important roles in growth, metabolism, and tissue maintenance (Rommel et al., 2001; Glass, 2005). However, precise regulation of this system is required, as both insufficient and excessive signaling can be harmful (Richardson et al., 2004; Junnila et al., 2013). In skeletal muscle, IGF-1 influences protein synthesis, regeneration, and metabolic function, establishing it as a central pathway linking physical performance to long-term health outcomes (Barclay et al., 2019). The present findings suggest that low and high intrinsic exercise capacity are accompanied by distinct patterns of IGF-1–JAK2–STAT5B signaling in skeletal muscle (Chaudhari et al., 2017), potentially contributing to divergent aging trajectories. Dysregulation of this pathway has been associated with insulin resistance, sarcopenia, and metabolic diseases, while proper regulation has been linked to the maintenance of muscle function and an extended lifespan (Friedrich et al., 2012; Junnila et al., 2013; Ascenzi et al., 2019; Barclay et al., 2019). Therefore, altered IGF-1-related signaling in low exercise capacity phenotypes may be a molecular pathway through which reduced exercise capacity leads to increased disease risk. Emerging evidence suggests that clock genes interact with the IGF-1 signaling pathway (Chaudhari et al., 2017; Jordan et al., 2017), which indicates the existence of a coordinated regulatory network rather than independent mechanisms. Thus, these findings suggest potential associations between selected clock gene transcript expression and IGF-1–related signaling markers in skeletal muscle that may be relevant to intrinsic exercise capacity and aging-related phenotypes.

Interestingly, expression of the muscle atrophy–related genes *MuRF-1* and *Atrogin-1* did not differ significantly between HCR and LCR rats despite substantial differences in intrinsic aerobic capacity. This observation may suggest that reduced exercise capacity in aged LCR rats is not primarily driven by overt activation of canonical muscle atrophy pathways. Instead, the observed phenotype differences may be more closely associated with alterations in metabolic regulation, anabolic signaling balance, or skeletal muscle functional quality rather than increased expression of ubiquitin–proteasome–related atrophy markers. These findings are consistent with the concept that aging-related declines in muscle function and aerobic performance can occur independently of pronounced activation of classical atrophy signaling pathways (Joseph et al., 2012; Cruz-Jentoft et al., 2019).

### 4.3 Implications for exercise medicine and healthy aging

The identification of clock gene and IGF-1–related pathways as molecular correlates of intrinsic exercise capacity may have implications for exercise physiology and aging research. Skeletal muscle represents a physiologically relevant tissue in which genetic background, circadian regulation, and metabolic signaling converge (Pedersen and Febbraio, 2008; Hawley et al., 2014). A better understanding of these molecular features may contribute to refining strategies aimed at optimizing exercise interventions across different levels of aerobic capacity. In addition, the present findings raise the possibility that circadian and growth-related pathways participate in the regulatory landscape associated with intrinsic exercise capacity. Although the current study does not establish therapeutic targets, these pathways may warrant further investigation in the context of aging and metabolic health. Such exploration could be particularly relevant for populations with reduced exercise tolerance, including older individuals or those with metabolic disorders.

### 4.4 Limitations and future directions

Several limitations should be acknowledged. First, the cross-sectional nature of the present study precludes causal inference regarding the relationships among skeletal muscle clock gene expression, IGF-1–related signaling, and intrinsic exercise capacity. In addition, the present study focused specifically on skeletal muscle, despite the well-established contributions of other metabolically active tissues, including liver, adipose tissue, and central regulatory systems, to systemic exercise capacity and metabolic regulation. Therefore, the present findings should be interpreted as skeletal muscle–associated molecular signatures rather than evidence of integrated systemic regulation. Second, the present study focused primarily on molecular markers and did not directly assess functional outcomes such as mitochondrial respiration, muscle contractile performance, or broader endocrine and metabolic responses. In addition, downstream anabolic signaling pathways, including PI3K-AKT-mTOR signaling, as well as skeletal muscle–derived secretory factors and myokines, were not evaluated. These analyses may provide additional mechanistic insight into the relationship between intrinsic exercise capacity, skeletal muscle signaling, and systemic metabolic regulation. Third, clock gene expression was assessed at a single circadian time point (ZT2–ZT3), which limits interpretation regarding circadian rhythmicity, phase shifts, or amplitude-dependent regulation. Therefore, the observed differences in *Cry1* and *Bmal1* transcript expression may reflect altered circadian alignment rather than baseline expression differences. Fourth, the present study was observational in nature and did not employ pharmacological inhibitors or genetic models targeting JAK2- or IGF-1–related signaling pathways. Accordingly, the present findings do not establish causal mechanistic relationships between altered signaling balance and intrinsic exercise capacity. Finally, only aged female rats were included in the present study, which may limit generalizability to males and younger populations. Future studies incorporating longitudinal designs, multiple circadian sampling time points, multi-tissue analyses, mechanistic interventions, and sex-specific comparisons will be important for further clarifying the molecular mechanisms linking intrinsic exercise capacity, skeletal muscle regulation, aging, and metabolic health.

## 5 Conclusion

In conclusion, the present study indicates that intrinsic exercise capacity is associated with distinct skeletal muscle molecular signatures, including differences in the expression of selected clock gene transcripts measured at ZT2–ZT3 and alterations in IGF-1–related signaling balance. These findings provide additional insight into the relationships among aerobic capacity, aging, and disease susceptibility, and highlight skeletal muscle as a physiologically relevant tissue in which intrinsic biological differences may be reflected in long-term health outcomes.

## Data Availability

The original contributions presented in the study are included in the article/**Supplementary Material**. Further inquiries can be directed to the corresponding author.
