# Supplementary material for: Intrinsic exercise capacity is associated with skeletal muscle clock gene and IGF-1 signaling in aged low- and high-running capacity rats
Source: Front Physiol. 2026 Jun 9;17:1818866. doi: 10.3389/fphys.2026.1818866 (PMC13286783; doi:10.3389/fphys.2026.1818866)
Supplement: Supplementary file 1 [file DataSheet1.docx]

Supplementary Material

# Supplementary Data

Supplementary Material should be uploaded separately on submission. Please include any supplementary data, figures and/or tables.

Supplementary material is not typeset so please ensure that all information is clearly presented, the appropriate caption is included in the file and not in the manuscript, and that the style conforms to the rest of the article.

# Supplementary Figures and Tables

For more information on Supplementary Material and for details on the different file types accepted, please see [here](https://www.frontiersin.org/guidelines/author-guidelines#supplementary-material).

## Supplementary Figures

**
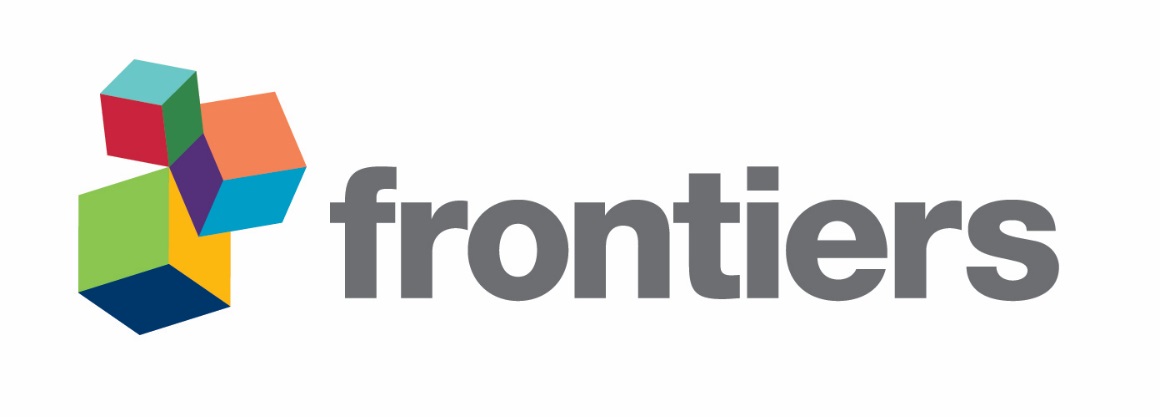
**

**Supplementary Figure 1.** The figure legends are required to have the same font as the main text, 12 point normal Times New Roman, single spaced. Please use a single paragraph for each legend and prepare the figures keeping in mind the PDF layout.

**Supplementary Figure 1**. Body weight of HCR and LCR rats.

Body weight measurements in high-capacity runner (HCR) and low-capacity runner (LCR) rats at the time of experimentation. Data are presented as individual values with mean ± SD. Statistical comparisons between groups were performed using unpaired two-tailed Student’s t-tests. *p < 0.05 versus HCR.
